# The Effect of Dietary Supplementation with Spent Cider Yeast on the Swine Distal Gut Microbiome

**DOI:** 10.1371/journal.pone.0075714

**Published:** 2013-10-09

**Authors:** Aditya Upadrasta, Lisa O’Sullivan, Orla O’Sullivan, Noel Sexton, Peadar G. Lawlor, Colin Hill, Gerald F. Fitzgerald, Catherine Stanton, R. Paul Ross

**Affiliations:** 1 Teagasc Food Research Programme, Moorepark, Fermoy, County Cork, Ireland; 2 Teagasc, Pig Production Development Unit, Moorepark Research Centre, Fermoy, County Cork, Ireland; 3 Department of Microbiology, University College Cork, Cork, Ireland; 4 Alimentary Pharmabiotic Centre, University College Cork, Cork, Ireland; 5 Cybercolors Ltd, Food Ingredients Company, Cork, Ireland; Wageningen University, Netherlands

## Abstract

**Background:**

There is an increasing need for alternatives to antibiotics for promoting animal health, given the increasing problems associated with antibiotic resistance. In this regard, we evaluated spent cider yeast as a potential probiotic for modifying the gut microbiota in weanling pigs using pyrosequencing of 16S rRNA gene libraries.

**Methodology and Principal Findings:**

Piglets aged 24–26 days were assigned to one of two study groups; control (n = 12) and treatment (n = 12). The control animals were fed with a basal diet and the treatment animals were fed with basal diet in combination with cider yeast supplement (500 ml cider yeast containing ∼7.6 log CFU/ml) for 21 days. Faecal samples were collected for 16s rRNA gene compositional analysis. 16S rRNA compositional sequencing analysis of the faecal samples collected from day 0 and day 21 revealed marked differences in microbial diversity at both the phylum and genus levels between the control and treatment groups. This analysis confirmed that levels of *Salmonella* and *Escherichia* were significantly decreased in the treatment group, compared with the control (P<0.001). This data suggest a positive influence of dietary supplementation with live cider yeast on the microbial diversity of the pig distal gut.

**Conclusions/Significance:**

The effect of dietary cider yeast on porcine gut microbial communities was characterized for the first time using 16S rRNA gene compositional sequencing. Dietary cider yeast can potentially alter the gut microbiota, however such changes depend on their endogenous microbiota that causes a divergence in relative response to that given diet.

## Introduction

The mammalian gastrointestinal tract (GIT) is among the most densely populated microbial ecosystems, with the colon harbouring a microbial load of ∼10^14^ cells/host [1]. This “virtual organ” plays a role in nourishment, epithelial cell development and regulation, and a switch to instruct the innate immunity [2]. The gut microbiome plays a major role in digestive physiology by assisting in nutrient absorption and assimilation processes, thereby maintaining homeostasis in the host gut. A balanced microbial composition is considered essential for host health [3] and disturbances to the healthy microbial community often results in a dysfunctional gut, leading to gut related disorders and abnormalities. The majority of the microbes that are detected in the GIT and other habitats are unculturable using routine culture methods. Various methods have been developed to overcome this hurdle based on 16s rRNA gene sequences, such as DGGE, TGGE ARDRA, T-RFLP, ITS typing, long-PCR-RFLP, SSCP and ARISA [4], which facilitate the identification of microbes residing in these complex ecosystems. Another widely accepted technique in microbial taxonomy research is 16s rRNA (small sub unit, SSU) gene based classification. Along with rapidly emerging metagenomic approaches, together with the application of the 16s rRNA gene amplicon pyrosequencing, it is now possible to decipher the proportions of both cultured and uncultured phylotypes present in any complex ecosystem. Such approaches have been used to study gut microbiota in obesity [5], diabetes in a rat model [6], the effect of a high zinc diet on pig ileal bacterial communities [7], a fibrous diet in dogs [8], and autoimmune development [9]. Moreover, the tracking of the gut microbiome of humans and animals provides a link between dietary habits and gut microbes. This link shapes the co-evolution of microorganisms with the evolution of their host and dietary patterns [10]. These studies favour the symbiosis concept and strengthen the host-microbe relationship, where there is a mutual benefit to both the host and the microbes to maintain homeostasis. These datasets can be used in the future to generate a “microbe atlas” with reference to particular diseases and healthy states, which can be used as potential microbial biomarkers. Although each host varies in terms of metabolism, geography and environment, the knowledge obtained from these studies can be used to address the questions about microbial life in a specific habitat, their functionalities and their co-evolution along with the host.

A wide variety of strains, such as lactobacilli, bifidobacteria and yeast have been exploited as probiotics in humans and animals. Probiotics are described as ‘live microorganisms which when administered in adequate amounts confer a health benefit on the host’ [11] and are considered as potential alternatives to antibiotics in veterinary medicine in some instances. There is also a food safety aspect to using probiotics in animal feed. Food borne pathogens are a major cause of illness as a result of the consumption of meat products, raw vegetables and dairy products processed and/or prepared in an unhygienic manner. *Salmonella* and *Campylobacter* species are the most commonly reported food borne pathogens in meat and dairy products, while some other pathogens, such as *Clostridium perfringens*, *E. coli* O157:H7, *Listeria, Arcobacter* and *Helicobacter* spp. can also occur. In 2001, approximately 15,500 cases of human salmonellosis and campylobacteriosis cases were reported in the European Union (EU) [12]. Previously, we had developed a five strain probiotic mixture of lactobacilli and pediococci and demonstrated that it could reduce *Salmonella* shedding in pigs [13]–[16].

Apart from lactobacilli and bifidobacteria as probiotic supplements, there has been increasing attention on yeast cultures and yeast products as feed additives in human and animal nutrition, although much of the early research concentrated on animal growth, weight gain and performance [17]–[19]. The application of yeast and yeast products as probiotics and their beneficial effects have been well-documented using different animal models and humans [20]–[27]. However the effect of feeding yeast as a dietary adjunct is still ambiguous. In a number of studies, dietary supplementation of live yeast, yeast cultures or yeast cell wall products have been reported to improve the growth performance in weanling pigs [21], [23], [25], [26], [28], [29], while others have reported no beneficial effects of feeding and supplementation of yeast on swine growth and performance [23], [25], [30]. The form in which probiotic yeast is administered is also an important consideration. For example, feeding liquid fermented yeast form as a dietary supplement improved animal performance, when compared to yeast fed in dry form. The liquid fermented diets also helped to maintain the intestinal integrity during post weaning periods, thereby reducing post-weaning diarrhoeal symptoms in pigs [31]. This study evaluated spent cider yeast as a dietary probiotic supplement for modifying gut microbiota in weanling pigs using compositional sequencing.

## Materials and Methods

### Animal Housing and Management

The pig-feeding trial was conducted under European Union Council Directive 91/630/EEC (outlines minimum standards for the protection of pigs) and European Union Council Directive 98/58/EC (concerns the protection of animals kept for farming purposes) and was approved by, and a license obtained from, the Irish Department of Health and Children. A total of 24 crossbred (Large White × Landrace) pigs were weaned at approximately 24 to 26 days of age. The pigs were fed a common starter diet (16.5 MJ/kg digestible energy (DE) and 16.5 g/kg lysine) for 7 days and a common link diet (15.5 MJ/kg DE and 15.0 g/kg lysine) for another 4 days after weaning. Composition of diets, fed to the pigs, during pre-trial and trial (control pigs) and treatment pigs was tabulated in Table S1.

Following this acclimatization period, pigs were blocked by litter origin, sex and weight and individual pigs were randomly assigned to one of two treatments: (1) control, fed only basal diet consisting of 15.5 MJ/kg DE and 15.5 g/kg lysine (n = 12) and (2) basal diet in combination with cider yeast supplement (500 ml providing on average of 7.6 log CFU/ml, n = 12). The spent cider yeast was obtained as a waste mass from the former apple cider fermentation. An average of 7.6 log CFU/ml live yeasts were recorded in the obtained mass for the feeding trial. The duration of the experiment was 21 days. For each pig, body weight and feed intake was recorded at four time points on days - 0, 7, 14 and 21 of the study. For the cider yeast supplemented group, consumption of cider yeast was measured daily between day 0 to day 21. Weekly consumptions (ml) of cider yeast was converted to a meal equivalent (g) as follows:

Cider yeast consumption (ml)×0.15)/0.87 = meal equivalent cider yeast (g), where 0.15 = proportion of dry matter in the cider yeast and 0.87 is the proportion of dry matter in a normal pig diet. Therefore, feed intake of the pigs on cider yeast diet was calculated as feed disappearance of basal diet+meal equivalent intake of cider yeast (g). Each pig was individually housed in fully slatted pens (1.07 m×0.06 m) with plastic slats (Faroex, Manitoba, Canada) in a total of 3 rooms with 8 pens per room (4 pigs/treatment/room). Each pen had a door mounted stainless steel trough (410 mm long) with a divider in the middle. The left compartment of each trough was used for feeding cider yeast, while the right compartment used for dry pelleted feed (control diet) to which the pigs were given *ad-libitum* access. Feed intake was measured as the disappearance of dry pelleted feed for the control group and the disappearance of dry pelleted feed plus the fresh weight meal equivalent of cider yeast for the experimental group. Room temperature was maintained at 28–30°C in the first week and reduced by 2°C per week to 22°C in the fourth week.

### Faecal Sampling, DNA Extraction, PCR and Pyrosequencing

At time points day 0, and 21 freshly voided faeces was collected from the pigs. Approximately 230–300 mg faecal material was weighed and stored immediately at −20°C prior to DNA extractions. Total metagenomic DNA was extracted from individual feacal samples using the QIAamp DNA Stool Mini Kit (Qiagen, West Sussex, UK) according to manufacturer’s instructions. The microbial composition of these samples was evaluated by pyrosequencing of 16S rRNA tags (V4 region: 239 nucleotide long) amplified using universal 16S primers predicted to bind 94% of all 16S genes that is the forward primer F1 (5′-AYTGGGYDTAAAGNG-3′) and a combination of four reverse primers R1 (5′-TACCRGGGTHTCTAATCC-3′), R2 (5′- TACCAGAGTATCTAATTC-3′), R3 (5′-CTACDSRGGTMTCTAATC-3′) and R4 (5′-TACNVGGGTATCTAATC-3′) [32]. The primers incorporated the proprietary 19-mer sequences at the 5′-end to allow emulsion-based clonal amplification for the 454 pyrosequencing system. Unique molecular identifier (MID) tags were incorporated between the adaptamer and the target-specific primer sequence, to allow identification of individual sequences from pooled amplicons. Amplicons were cleaned using the Qiagen PCR purification kit (Qiagen, West Sussex, UK) and sequenced on a 454 sequencer FLX Titanium platform (MWG, Ebersberg, Germany) according to 454 protocols.

### Sequence Processing and Analysis

The sequences from faecal DNA samples of 16 animals (8 control and 8 treatment) at two time points (day 0 and day 21) were processed and analyzed to determine differences at all taxonomic and community levels using PANGEA (Pipeline for Analysis of Next GEneration Amplicons) [33]. Since the data for one animal at day 0 is not available there are 15 animals in day 0, 8 animals for the control and 8 animals for the treatment group at day 21. In PANGEA small sequences (<100) are discarded, poor quality (phred quality score <20) ends are trimmed, 16S rRNA gene sequences are separated by representative barcode, and the closest cultured relative member of each sequence is identified using MEGABLAST [34] against a modified bacterial RDP-II database prepared using Taxcollector downloaded on Nov 2010 [35]. The significant differences of taxa (Phylum, Class, Order, Family, Genus and Species) between control and treatment animals are determined using a modified χ^2^-test which includes a false discovery rate (fdr) determination to get a P-value for the null hypothesis. The unclassified sequences were clustered with a sequence identity threshold at 0.8 similarities to Domain/Phylum, 0.9 to Class/Order/Family, 0.95 to Genus and 0.99 to the Species level. In order to quantitatively estimate the microbial diversity, the reads were normalized to the number of reads in the sample which had the smallest number of reads. Qualitative analysis is performed with the unnormalized reads. In order to evaluate the similarities and differences in the diversity of microbial communities between the groups, FastUnifrac [36] was performed using the default options in QIIME using the QIIME virtual machine 1.1.0 [37]. A beta diversity distance matrix is computed by Qiime which is then used to build the unweighted pair group method with arithmetic mean (UPGMA) tree. This tree is visualised using FigTree [38]. To validate the UPGMA results, Jackknifing analysis is also performed and the results presented. For UPGMA clustering, a constant random number of sequences are selected from each animal to generate an UPGMA tree which is compared to the UPGMA tree built from all the animals using 100 permutations to generate the tree nodes. Jackknifing is performed with 700 sequences randomly selected from the animals using 100 permutations to get the Jackkniffe support values. The same database (Taxcollector) used in PANGEA is also used for all the Qiime analysis. Sample richness is calculated for all animals in day 0, control animals in day 21 and treatment animals in day 21. In order to provide a better understanding on how dietary cider yeast affects the diversities of microbial populations in each pig gut microbiome, sample richness analysis was also performed on each individual animal at day 0 and day 21. This was performed as follows: the sequences from each animal were aligned using MUSCLE [39] and the aligned sequences were used to generate a distance matrix using the dnadist subroutine in the PHYLIP package [40]. This distance matrix is then read using MOTHUR [41] and the statistical quantities are calculated. The changes in the percentage of relative abundance values were calculated from the number of occurrences in the control group (N_c21_) and the number of occurrences in the treatment group (N_t21_) using the following equation [33].



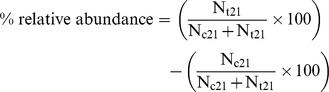



Sequence reads obtained from this study are available from the Sequence Read Archive (SRA) (http://www.ncbi.nlm.nih.gov/sra), under study accession number SRP028111.

## Results

### Animal Performance

The average daily weight gain (ADG), daily feed intake (DFI) and feed conversion efficacy (FCE) for the control and treatment animals are listed in Table S2, together with SEM and p-values. The total consumption of cider yeast (ml) for the treatment pigs was 2171 (SD = 164.2 g), 2461 (SD = 192.2 g), 3069 (SD = 442.8 g) and 13963 (SD = 2386.9) during days 0–7, 7–14, 14–21 and 0–21, respectively. The total meal equivalent consumption of cider yeast for treatment pigs was 374 (SD = 28.3 g), 476 (SD = 33.1 g), 529 (SD = 76.3 g) and 1380 (SD = 125.3 g) during days 0–7, 7–14, 14–21 and 0–21, respectively. This was added to the disappearance of basal diet to calculate daily feed intake for treatment pigs (Table S3).

The control group had a higher daily feed intake (P<0.05) and higher average daily gain (P<0.001) during the period from day 7 to 14. Feed conversion efficiency was less for the control group compared with the experimental group (P<0.05) during the period from day 0 to 7 and less for the experimental group than the control group during the period from day 7 to 14 (P<0.001). Overall, the average live weight of the piglets was unaffected by the cider yeast treatment during all stages of the trial.

### Viable Yeast Counts in the Feed

The cider yeast feeding supplement fed daily contained 5.2×10^7^ log CFU/ml of total viable yeast and ∼1×10^10^ log CFU yeast was ingested daily by each animal throughout the trial. The pigs were fed for 21 consecutive days and sampled at both day 0 and day 21- at which points the diversity of the faecal microbiota was analysed.

### Microbial Compositional Analysis by Pyrosequencing

A total of 139,072 sequences that passed the quality check were considered for further analysis. These sequences were classified to the genus level at a 95% sequence identity threshold. We analysed the percentages of sequences thus classified along with the non-parametric richness estimates for both groups and for individual animals in each group. The diversities and their abundance statistics are presented in Table S4.

### Population Dynamics from Phylum to Species Level

#### Phylum level

**Figure 1:**
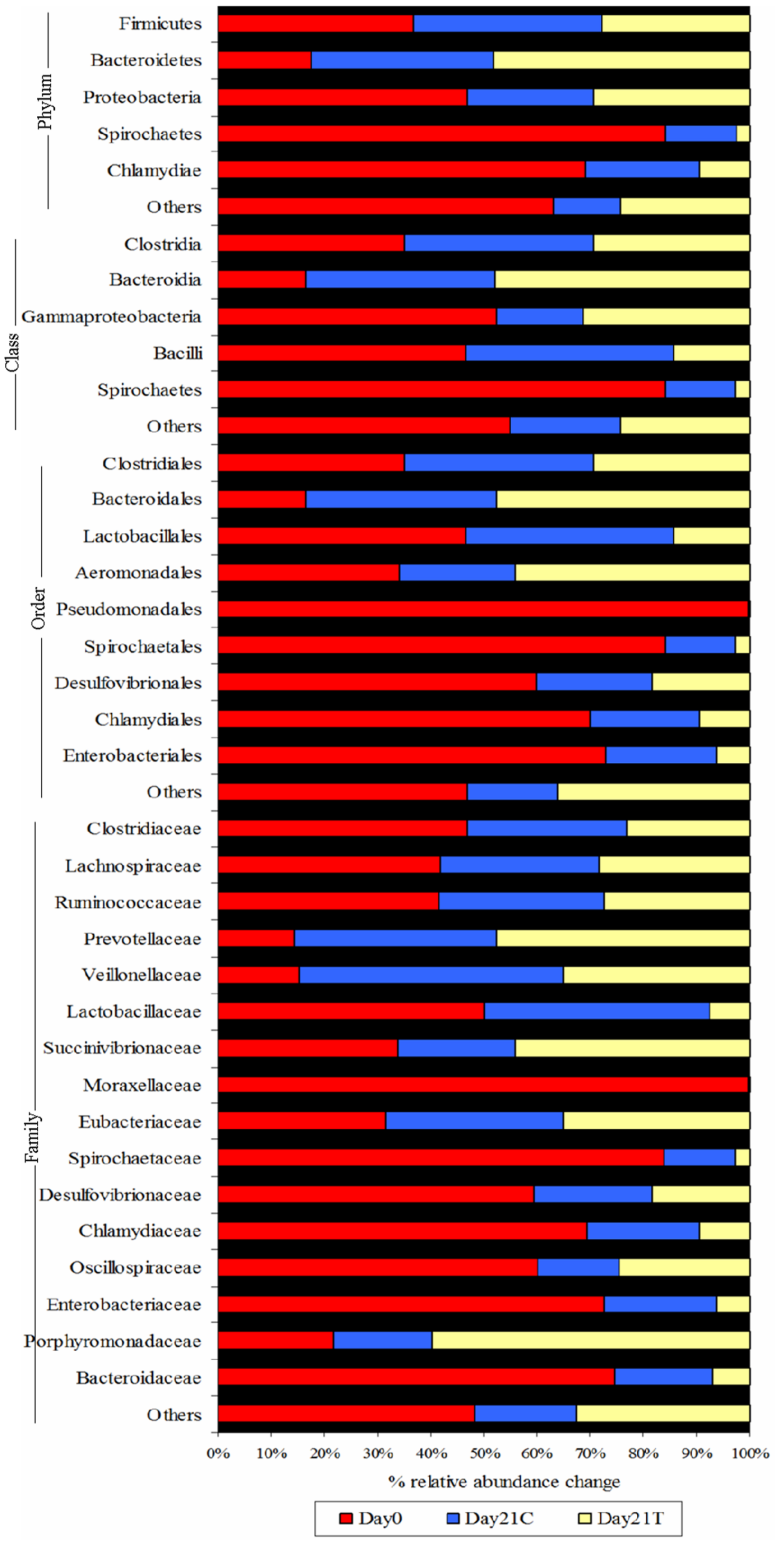
Percentage relative abundance of OTUs observed at the phylum, class, order and family levels in the pig distal gut microbiota at day 0, day 21 C (control) and day 21 T (treatment-cider yeast supplemented) groups.

From the total number of sequences at day 0, day 21 control and day 21 treatments, 87.9%, 88.3% and 84.5% of the sequences were assigned to the phylum level (Figure S1). The day 0 communities were dominated by members of the Firmicutes phylum (63.0%) when compared to the other phyla; Bacteroidetes (15.4%), Proteobacteria (14.8%), Spirochaetes (4.3%) and Chalmydiae (1.7%). At day 21, the relative abundance of Firmicutes remained unchanged in the control group at 60.74%, whereas they were significantly lower (P<0.01) in the treatment group at 47.6%. The second most abundant phylum, the Bacteroidetes, had increased from 15.4% at day 0 to 30.4% for the control and 42.4% for the treatment group on day 21. A total of 15 phyla were found in all animals at day 0 (Figure 1). Apart from the five major phyla, ten other phyla were also observed which accounted for only 0.57% of the total reads. The differences in abundance of Firmicutes, Bacteroidetes, Proteobacteria, Spirochaetes and Chlamydiae observed between treatment and control groups at day 21 were significant (P<0.01) as determined by the modified χ^2^-test shown in Table S5.

#### Class and order level

Similar trends were observed between communities at the level of Class (Figure 1). At day 0 the three most abundant classes were the clostridia (57.1%), bacteroidia (13.8%) and γ-proteobacteria (12.8%). Clostridia levels remained unchanged at day 21 in the control group, whereas they decreased from 57.1% to 47.6% in the treatment animals over 21 days. Bacteroidia levels increased over time from 13.8% to 29.7% in the control group and to 39.8% in the treatment group at day 21. Similarly, γ-proteobacteria increased in the control and treatment animals compared with the day 0 time point. Similar trends were observed at the order level. Clostridiales sequences decreased to 47.6% in the treatment group when compared to day 0 (57.1%) and day 21 controls (57.9%). Bacteroidales increased over time from day 0 (13.8%) to day 21 in controls (29.7%) and treatments (39.8%). In contrast, Lactobacillales sequences declined over time from 6.4% at day 0 to 5.3% and 1.9% in controls and treatments at day 21.

#### Family level

At the family level, three families among the Firmicutes phylum, Clostridiaceae, Lachnospiraceae, and Ruminococcaceae were more abundant at day 0 and gradually decreased in control and treatment animals at day 21 (Figure 1). In contrast, Viellonellaceae, which also belongs to the Firmicutes phylum, increased from day 0 to day 21 in control and treatment animals, but decreased in treatments compared to controls at day 21. The Prevotellaceae family, from the Bacteriodetes phylum, significantly increased (P<0.01) from day 0 to day 21 for both groups, but was slightly higher (P<0.05) in day 21 treatments than day 21 controls. Notably, the percentage relative abundance change in Enterobacteriaceae (69%) in cider yeast supplemented group (P<0.001) compared to the control group on day 21.

#### Genus level

**Figure 2:**
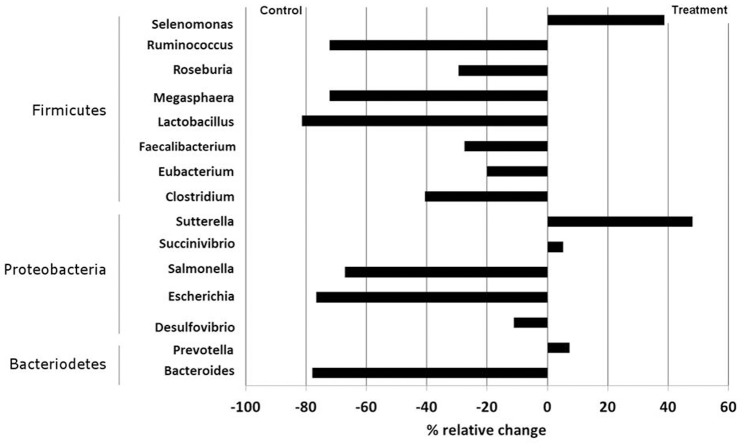
Percentage of relative abundance change at genus level in the control and treatment animals at day 21 (P<0.001).

A 95% identity level was applied to classify the sequences at the genus level. From the total number of sequences 44%, 53% and 49% of sequences were taxonomically assigned at day 0, day 21 control and day 21 treatments, respectively (Figure S1). There was no consistency observed at the genus level in any of the three groups. At day 0, *Clostridium* and *Lactobacillus* genera belonging to the Firmicutes phylum were most abundant, followed by *Prevotella*, *Acinetobacter* and *Ruminococcus* belonging to the phyla Bacteriodetes, Proteobacteria and Firmicutes, respectively. In order to get a clearer understanding of the variation between the control and treatment groups at day 21, the percentage of relative abundance was plotted for the genera that were significantly different (P<0.001) (Figure 2).

*Prevotella* members were increased at day 21 in both groups, with greater increase found in treatments than controls (Figure 2). In addition, pathogenic genera such as *Salmonella* and *Escherichia* numbers (P<0.001) were reduced in the cider yeast supplemented group compared to the control group (Figure 2) (for p-values including false discovery rates (fdr) between cider yeast supplemented and control diet animals at day 21 see Table S5). Very few sequences at day 0 were assigned to the genus *Bifidobacterium* (0.006%), which belonged to the Actinobacteria phylum, whereas no bifidobacteria were observed in either group at day 21.

#### Species level

A total of 196, 125 and 99 phylotypes were observed at day 0, day 21 controls and treatments respectively. At the 99% similarity level, 12.5%, 15.4% and 12.0% of day 0, day 21 control and day 21 treatment sequences were classified to known cultured species. At day 0, *Acinetobacter* sp., belonging to the γ-proteobacteria phylum, were the most prevalent species at 9.5%, however, this species was absent in cider yeast supplemented and control diet groups at day 21. With regard to *Lactobacillus*, the sequences assigned to the species level belonged to *L*. *amylovorus*, *L*. *reuteri* and *L*. *johnsonii*. At day 0, *L*. *amylovorus* and *L*. *reuteri* were present at higher numbers when compared to day 21 control group. Furthermore, relative percentages of *L*. *johnsonii* increased from day 0 (2.5%) to day 21 in the control group (4.3%) and decreased in the cider yeast (2%) supplemented group. Similarly, a significant decrease (P<0.05) in the plate counts of total numbers of *Lactobacillus* sp. observed in the cider yeast supplemented group compared to the control group at day 21. An increase in numbers of butyrate producing organisms, such as *Faecalibacterium* spp., *Faecalibacterium prausnitzii* (P<0.05) was observed in the cider yeast supplemented group. The percentage relative abundance change in enteric pathogens, such as *Salmonella enterica* (63%) and *Escherichia fergusonii* (53%) was reduced in the cider yeast supplemented group (P<0.001) compared to the control group.

### UniFrac Distance Metrics, UPGMA and Jackknife Analyses

**Figure 3:**
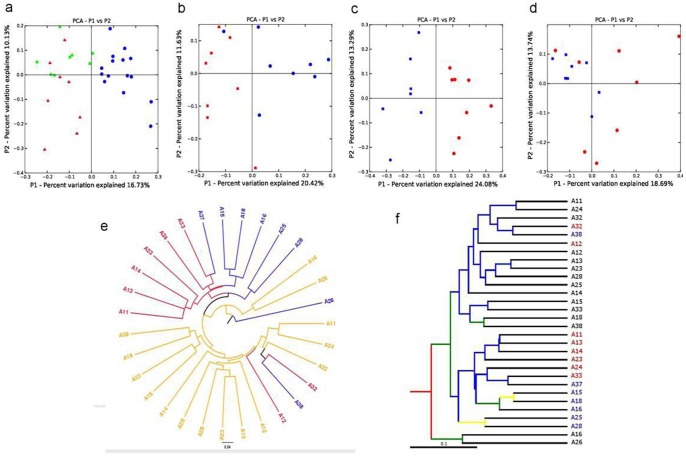
Unweighted principal component analysis. For: a) control and treatment animals in day 0 (blue), control animals in day 21 (green) and treatment animals in day 21 (red) b) control animals in day 0 (blue) and control animals in day 21 (red) c) treatment animals in day 0 (blue) and treatment animals in day 21(red) d) control animals in day 21 (blue) and treatment animals in day 21 (red). UPGMA clustering and Jackkniffing for the unweighted UniFrac data e) For the UPGMA cladogram on the left: Orange colour represents animals in day 0; red for the control animals in day 21 and blue for the treatment animals in day21. d) For the Jackknife supported tree layout the labels are coloured according to the group as: Black for animals in day 0; red for the control animals in day 21 and blue for treatment animals in day 21. The lines are coloured by the Jackknife supported percentages: Red for 75–100% support; Green for 50–75% support; Yellow for 25–50% support and Blue for <25% support.

The similarities and dissimilarities between the groups was evaluated by unweighted (based on presence or absence of taxa) and weighted (based on relative abundance) UniFrac based principal component analysis (PCA) (Figure 3 and Figure S2). In both UniFrac analyses, clustering was observed among the animals based on their diets. At day 0, all of the animals (n = 15) were clustered together and at day 21, the treatment animals clustered separately from day 0 animals (P<0.01). In order to understand the variation of the diet at the two time points, the control group animals at day 0 and day 21 were compared. A similar comparison was performed for the treatment group at day 0 and day 21 and the separation between the clusters was more evident in this case. A cladogram generated using UPGMA clustering of day 0, day 21C and day 21T animals showed a distinct clustering by diets (Figure 3e & Figure S3) and the Jackknife clustering of groups showed (Figure 3f & Figure S3a, b) clustering (≥75% on most of the nodes) by dietary treatment.

### Biodiversity and Species Richness

Group-based and individual animal based rarefaction curves and non-parametric richness estimations were calculated. Rarefaction curves for the number of OTU_0.03_ observed for each group on day 0 (n = 15), day 21 controls (n = 8) and day 21 treatments (n = 8) and for each individual animal is given in the supporting information (SI) (Figures S4a, b, c, d). Estimated Good’s coverage was 99% for the pooled groups and ranged from 80 to 99% for each individual animal in the groups, indicating the level of sequence coverage was adequate. Sample richness estimators Chao1, ACE and Simpson indices from MOTHUR and the normalized and unnormalized Shannon indices from PANGEA for the OTUs classified at the genus level (95% identity) are presented in the Table S4. The unnormalized Shannon indices for most of the animals ranged 4 to 5 with a few exceptions. Since Shannon index is a ratio of the number of OTUs classified to the total number of sequences in a sample, the exact values are not informative unless the total number of sequences is normalised. Since the number of sequences from each sample was similar, the normalised Shannon indices can be used to compare the number of classified OTUs in the different samples. These values are approximately 4, 3, and 2 for the animals at day 0, for the control and treatment animals at day 21 respectively. This suggests that there is a decrease in the diversity in the animals at day 21 compared to day 0 and that the diversity is even lower in the treatment group than in the control group at day 21. These values are consistent with the observations made with the relative abundance values at each taxonomic level shown in Figure 3. The other statistical analyses non-parametric richness estimator’s Simpson indices and Abundance Coverage Estimator (ACE) data also reflect this trend (Table S4). The microbial community appeared more diverse at day 0, when compared to the treatment group at day 21.

## Discussion

This study evaluated the impact of dietary supplementation of spent cider yeast on porcine distal gut microbial communities. We found that cider yeast as a dietary adjunct had no discernable effect on the host physiology as inferred from the feed intake and growth performance results. Similarly feeding live yeast cells and yeast cell wall products did not affected the ADFI and ADG [24]. However, there were significant changes in the proportions of bacterial communities observed at all OTU levels between the cider yeast supplemented group and control diet group at day 21. In particular, the effects of dietary cider yeast on four major phyla: Proteobacteria, Actinobacteria, Bacteriodetes and Firmicutes in the distal gut were analysed. The percentage of Proteobacteria increased in the group fed with cider yeast diet compared to the control diet. However, the effect of cider yeast appears to be selective and among the proteobacteria, the population of enteric pathogens such as *Salmonella* and *Escherichia* decreased significantly (Figure 2).

Lactobacilli counts were reduced in the cider yeast supplemented group compared with the control (P<0.05). The outer layer of the yeast cell wall is composed of mannose associated protein called mannan and mannose oligosaccharide which may function as prebiotic components. Similarly, in a study conducted on humanized microbiome mouse models (HGM) it was shown that the mannose oligosaccharide which functions as a prebiotic facilitating the increase in bifidobacteria might also cause a reduction in the lactobacilli counts since the lactobacilli numbers were observed to diminish upon prebiotic administration [42]. It has been reported that supplementation of inulin-type fructans to the diet or drinking water resulted in less diarrhoeal occurrence, reduced mortality and pathogen shedding in animals [43].

As for the Firmicutes phylum, an abundance of *Faecalibacterium* spp., *Faecalibacterium prausnitzii* (P<0.05), suggests a healthy symbiotic association between yeast cells and their cell wall oligosaccharides. Recently, the possible probiotic attributes of *Faecalibacterium prausnitzii* was postulated in murine colitis models, such as anti-inflammatory and immune modulatory effects [44]. A strong positive correlation between the numbers of *Faecalibacterium prausnitzii* and high levels of faecal butyrate was observed in healthy human subjects [45]. Firmicute bacteria related to *Ruminococcus* spp., were abundant in the control and treatment groups. The *Roseburia hominis* numbers increased in a response to the cider yeast. The *Roseburia* spp., *Eubacterium* spp., and their closely related bacterial groups are known to contain amylolytic species and known butyrate producers [46]. Moreover, the other families Eubacteriaceae and Porphyromonadaceae were abundant in the treatment group and the members of these families can metabolize complex sugars and produce lactate and butyrate as end products [47].

Although diversity estimates based on OTUs may differ amongst the individual animals, significant perturbations were observed in the porcine GI microbiota according to dietary treatments. Each individual animal was evaluated using the unweighted and weighted UniFrac and UPGMA clustering and the animals were clustered according to their dietary treatments (Figure 3). Thus the PCA and UPGMA analysis supports the dietary pattern at the end of day 21.

16S rRNA sequence-based comparisons of human [1], [2], [48]–[50], swine [7] and canine [8] faecal microbiota have revealed high levels of inter-individual variations. The Shannon indices obtained for these animals using normalized reads show a substantial increase in the sequencing tags leading to higher diversity indices. However, the gut microbial composition also varies in each individual [51] and also depends on the host’s response to the given diet. Dietary cider yeast can potentially alter the gut microbiota, however such changes depends on their endogenous microbiota which may cause a divergence in relative response to that given diet.

### Conclusions

The present study suggests that dietary cider yeast has the potential to be used as a supplement for enhanced gut function and the reduction of *Salmonella* carriage in pigs. Cider yeast has the potential to selectively inhibit the enterobacterial (such as *Salmonella* spp., and *Escherichia* spp.,) populations. Consequently, cider yeast has the potential to serve as dietary supplement in animal nutrition to improve health status and to reduce the potential for zoonotic diseases. More robust studies are required with more animals, faecal fatty acid estimations and their bacterial community proportions of individual subjects, which can reveal the interactions between the diet and bacterial communities. Such studies would unravel the inter-play between diet-mediated alterations in bacterial secondary metabolites and their symbiotic relationships, which can make such studies more meaningful and therefore contribute in the development of new health related nutrition strategies.

## Supporting Information

Figure S1 **Percentages of sequences that are classified into OTU’s, using identity threshold of 80% for Phylum, 90% for Class, Order and Family, 95% for Genus and 99% for Species.**
(TIF)

Figure S2 **Weighted UniFrac principal component analysis.** for **a**) control and treatment animals in day 0 (blue-circles), control animals in day 21 (green-squares) and treatment animals in day 21 (red-triangles) **b**) Control animals in day 0 (red-squares) and control animals in day 21 (blue-circle) **c**) Treatment animals in day 0 (blue-squares) and treatment animals in day 21 (red-circles) **d**) Control animals in day21 (blue-squares) and treatment animals in day 21 (red-circles).
(TIF)

Figure S3 **a)** UPGMA clustering and Jackknifing for the weighted UniFrac data. For the UPGMA cladogram on the left: Orange colour represents animals at day 0; red for the control animals at day 21 and blue for the treatment animals at day 21. **b)** For the Jackknife supported tree layout the labels are coloured according to the group as: Black for animals in day 0; red for the control animals in day 21 and blue for treatment animals in day 21. The lines are coloured by the Jackknife supported percentages: Red for 75–100% support; Green for 50–75% support; Yellow for 25–50% support and Blue for <25% support.
(TIF)

Figure S4 **a)** Rarefaction curves for the animals in day 0 (blue), control animals in day 21 (orange) and treatment animal in day 21 (green) for the 0.03 distance uniqueness values. **b)** For the control animals and treatment animals at day 21 (D21). Animal labels are consistent with the labels used in the supplementary table ST3. **c)** For the control animals at day 0 and day 21 (A-animal, C-control D-day 0 or 21) **d)** For the treatment animals at day 0 and day 21 (A-animal, T-treatment D-day 0 or 21).
(TIF)

Table S1 **Composition of diets fed to the pigs.**
(DOC)

Table S2 **Effect of cider yeast on pig intake and growth performance.**
(DOC)

Table S3 **Amount of Cider yeast (ml) consumption by the animals during 21 days and its meal equivalent (g).**
(DOC)

Table S4 **Sample richness estimators, Shannon diversity indices, Chao1 richness, and Good’s coverage for the sequences classified at 95% level of similarity.** Shannon indices for the samples estimated from normalized and unnormalized sequences from PANGEA.
(DOC)

Table S5 **Comparison of taxonomic groups between the treatment (CY) and control groups at day 21.** Using modified Chi-square test with false discovery rate (FDR).
(DOC)

## References

[1] Gill SR, Pop M, DeBoy RT, Eckburg PB, Turnbaugh PJ, et al (2006) Metagenomic Analysis of the Human Distal Gut Microbiome. Science 312: 1355–1359. 16741115 10.1126/science.1124234 PMC3027896

[2] Eckburg PB, Bik EM, Bernstein CN, Purdom E, Dethlefsen L, et al (2005) Diversity of the Human Intestinal Microbial Flora. Science 308: 1635–1638. 15831718 10.1126/science.1110591 PMC1395357

[3] Les Dethlefsen MMFN, Relman DA (2007) An ecological and evolutionary perspective on human–microbe mutualism and disease. Nature 449: 811–818. 17943117 10.1038/nature06245 PMC9464033

[4] Rajendhran J, Gunasekaran P (2011) Microbial phylogeny and diversity: Small subunit ribosomal RNA sequence analysis and beyond. Microbiological Research 166: 99–110. 20223646 10.1016/j.micres.2010.02.003

[5] Ley RE, Bäckhed F, Turnbaugh P, Lozupone CA, Knight RD, et al (2005) Obesity alters gut microbial ecology. Proceedings of the National Academy of Sciences of the United States of America 102: 11070–11075. 16033867 10.1073/pnas.0504978102 PMC1176910

[6] Roesch LFW, Lorca GL, Casella G, Giongo A, Naranjo A, et al (2009) Culture-independent identification of gut bacteria correlated with the onset of diabetes in a rat model. ISME J 3: 536–548. 19225551 10.1038/ismej.2009.5 PMC2972309

[7] Vahjen W, Pieper R, Zentek J (2010) Bar-Coded Pyrosequencing of 16S rRNA Gene Amplicons Reveals Changes in Ileal Porcine Bacterial Communities Due to High Dietary Zinc Intake. Appl Environ Microbiol 76: 6689–6691. 20709843 10.1128/AEM.03075-09 PMC2950476

[8] Middelbos IS, Vester Boler BM, Qu A, White BA, Swanson KS, et al (2010) Phylogenetic Characterization of Fecal Microbial Communities of Dogs Fed Diets with or without Supplemental Dietary Fiber Using 454 Pyrosequencing. PLoS ONE 5: e9768. 20339542 10.1371/journal.pone.0009768 PMC2842427

[9] Giongo A, Gano KA, Crabb DB, Mukherjee N, Novelo LL, et al.. (2010) Toward defining the autoimmune microbiome for type 1 diabetes. The ISME Journal. 10.1038/ismej.2010.92 PMC3105672 20613793

[10] Ley RE, Hamady M, Lozupone C, Turnbaugh PJ, Ramey RR, et al (2008) Evolution of Mammals and Their Gut Microbes. Science 320: 1647–1651. 18497261 10.1126/science.1155725 PMC2649005

[11] FAO/WHO (2001) FAO/WHO, Evaluation of health and nutritional properties of probiotics in food including powder milk with live lactic acid bacteria. Report from FAO/WHO expert consultation 1–4 October: 1–4 October.

[12] Cavitte JC (2001) Present and future control of foodborne pathogens in poultry; revision of the European Community legislation on zoonoses. 46–58.

[13] Casey PG, Casey GD, Gardiner GE, Tangney M, Stanton C, et al (2004) Isolation and characterization of anti-*Salmonella* lactic acid bacteria from the porcine gastrointestinal tract. Letters in applied microbiology 39: 431–438. 15482434 10.1111/j.1472-765X.2004.01603.x

[14] Casey PG, Gardiner GE, Casey G, Bradshaw B, Lawlor PG, et al (2007) A Five-Strain Probiotic Combination Reduces Pathogen Shedding and Alleviates Disease Signs in Pigs Challenged with *Salmonella enterica* Serovar Typhimurium. Applied and Environmental Microbiology 73: 1858–1863. 17261517 10.1128/AEM.01840-06 PMC1828830

[15] Gardiner GE, Casey PG, Casey G, Lynch PB, Lawlor PG, et al (2004) Relative ability of orally administered *Lactobacillus murinus* to predominate and persist in the porcine gastrointestinal tract. Applied and Environmental Microbiology 70: 1895–1906. 15066778 10.1128/AEM.70.4.1895-1906.2004 PMC383152

[16] Walsh MC, Gardiner GE, Hart OM, Lawlor PG, Daly M, et al (2008) Predominance of a bacteriocin-producing *Lactobacillus salivarius* component of a five-strain probiotic in the porcine ileum and effects on host immune phenotype. Fems Microbiology Ecology 64: 317–327. 18373687 10.1111/j.1574-6941.2008.00454.x

[17] Chapman JD (1988) Probiotics, acidifiers and yeast culture: a place for natural additives in pig and poultry production. 1988: 219–233.

[18] Kogan G, Kocher A (2007) Role of yeast cell wall polysaccharides in pig nutrition and health protection. Livestock Science 109: 161–165.

[19] Pearson V, Ewan RC, Zimmerman DR (1978) Energy Evaluation of a Yeast Single-Cell Protein Product for Young Pigs. Journal of Animal Science 47: 488–491.

[20] Line JE, Bailey JS, Cox NA, Stern NJ, Tompkins T (1998) Effect of yeast-supplemented feed on *Salmonella* and *Campylobacter* populations in broilers. Poultry Science 77: 405–410. 10.1093/ps/77.3.405 9521452

[21] Mathew AG, Chattin SE, Robbins CM, Golden DA (1998) Effects of a direct-fed yeast culture on enteric microbial populations, fermentation acids, and performance of weanling pigs. J Anim Sci 76: 2138–2145. 9734864 10.2527/1998.7682138x

[22] Martins FS, Rodrigues ACP, Tiago FCP, Penna FJ, Rosa CA, et al (2007) *Saccharomyces cerevisiae* strain 905 reduces the translocation of Salmonella enterica serotype Typhimurium and stimulates the immune system in gnotoblotic and convectional mice. Journal of Medical Microbiology 56: 352–359. 17314366 10.1099/jmm.0.46525-0

[23] van Heugten E, Funderburke DW, Dorton KL (2003) Growth performance, nutrient digestibility, and fecal microflora in weanling pigs fed live yeast. Journal of Animal Science 81: 1004–1012. 12723090 10.2527/2003.8141004x

[24] Van der Peet-Schwering CMC, Jansman AJM, Smidt H, Yoon I (2007) Effects of yeast culture on performance, gut integrity, and blood cell composition in weanling pigs. J Anim Sci: jas.2007-0110. 10.2527/jas.2007-0110 17609465

[25] White LA, Newman MC, Cromwell GL, Lindemann MD (2002) Brewers dried yeast as a source of mannan oligosaccharides for weanling pigs. Journal of Animal Science 80: 2619–2628. 12413084 10.2527/2002.80102619x

[26] Spring P, Privulescu M (1998) Mannanoligosaccharide: its logical role as a natural feed additive for piglets; Nottingham. Nottingham University Press. 553–561.

[27] Spring P, Privulescu M (1998) Mannanoligosaccharide: its logical role as a natural feed additive for piglets. Stamford: Alltech UK. 553–561.

[28] Li JY, Li DF, Gong LM, Ma YX, He YH, et al (2006) Effects of live yeast on the performance, nutrient digestibility, gastrointestinal microbiota and concentration of volatile fatty acids in weanling pigs. Archives of Animal Nutrition 60: 277–288. 16921925 10.1080/17450390600785343

[29] Rozeboom DW, Shaw DT, Tempelman RJ, Miguel JC, Pettigrew JE, et al.. (2005) Effects of mannan oligosaccharide and an antimicrobial product in nursery diets on performance of pigs reared on three different farms 1. Am Soc Animal Sci. 2637–2644. 10.2527/2005.83112637x 16230663

[30] Veum TL, Bowman GL (1973) *Saccharomyces cervisiae* Yeast Culture in Diets for Mechanically-Fed Neonatal Piglets and Early Growing Self-Fed Pigs. J Anim Sci 37: 67–71.

[31] Stein H (2007) Feeding the pigs, immune system and alternatives to antibiotics. 61801.

[32] Cole JR, Wang Q, Cardenas E, Fish J, Chai B, et al (2009) The Ribosomal Database Project: improved alignments and new tools for rRNA analysis. Nucl Acids Res 37: D141–145. 19004872 10.1093/nar/gkn879 PMC2686447

[33] Giongo A, Crabb DB, Davis-Richardson AG, Chauliac D, Mobberley JM, et al (2010) PANGEA: pipeline for analysis of next generation amplicons. ISME J 4: 852–861. 20182525 10.1038/ismej.2010.16 PMC2974434

[34] Zhang Z, Schwartz S, Wagner L, Miller W (2000) A Greedy Algorithm for Aligning DNA Sequences. Journal of Computational Biology 7: 203–214. 10890397 10.1089/10665270050081478

[35] Giongo A, Davis-Richardson AG, Crabb DB, Triplett EW (2010) TaxCollector: Modifying Current 16S rRNA Databases for the Rapid Classification at Six Taxonomic Levels. Diversity 2: 1015–1025.

[36] Hamady M, Lozupone C, Knight R (2009) Fast UniFrac: facilitating high-throughput phylogenetic analyses of microbial communities including analysis of pyrosequencing and PhyloChip data. ISME J 4: 17–27. 19710709 10.1038/ismej.2009.97 PMC2797552

[37] Caporaso JG, Kuczynski J, Stombaugh J, Bittinger K, Bushman FD, et al (2010) QIIME allows analysis of high-throughput community sequencing data. Nature methods 7: 335–336. 20383131 10.1038/nmeth.f.303 PMC3156573

[38] Rambaut A (2009) Fig Tree. V1.3.1 ed.

[39] Edgar RC (2004) MUSCLE: multiple sequence alignment with high accuracy and high throughput. Nucleic Acids Research 32: 1792–1797. 15034147 10.1093/nar/gkh340 PMC390337

[40] Felsenstein J (1989) PHYLIP-phylogeny inference package (version 3.2). Cladistics 5: 164–166.

[41] Schloss PD, Westcott SL, Ryabin T, Hall JR, Hartmann M, et al (2009) Introducing mothur: open-source, platform-independent, community-supported software for describing and comparing microbial communities. Applied and Environmental Microbiology 75: 7537. 19801464 10.1128/AEM.01541-09 PMC2786419

[42] Martin FPJ, Wang Y, Sprenger N, Yap IKS, Rezzi S, et al.. (2008) Top-down systems biology integration of conditional prebiotic modulated transgenomic interactions in a humanized microbiome mouse model. Molecular Systems Biology 4. 10.1038/msb.2008.40 PMC2516362 18628745

[43] Oli MW, Petschow BW, Buddington RK (1998) Evaluation of Fructooligosaccharide Supplementation of Oral Electrolyte Solutions for Treatment of Diarrhea (Recovery of the Intestinal Bacteria). Digestive Diseases and Sciences 43: 138–147. 9508515 10.1023/a:1018892524790

[44] Sokol H, Pigneur B, Watterlot L, Lakhdari O, Bermúdez-Humarán LG, et al.. (2008) Faecalibacterium prausnitzii is an anti-inflammatory commensal bacterium identified by gut microbiota analysis of Crohn disease patients. Proceedings of the National Academy of Sciences. 10.1073/pnas.0804812105 PMC2575488 18936492

[45] Benus RFJ, van der Werf TS, Welling GW, Judd PA, Taylor MA, et al (2010) Association between Faecalibacterium prausnitzii and dietary fibre in colonic fermentation in healthy human subjects. British Journal of Nutrition 104: 693–700. 20346190 10.1017/S0007114510001030

[46] Duncan SH, Hold GL, Barcenilla A, Stewart CS, Flint HJ (2002) Roseburia intestinalis sp. nov., a novel saccharolytic, butyrate-producing bacterium from human faeces. Int J Syst Evol Microbiol 52: 1615–1620. 12361264 10.1099/00207713-52-5-1615

[47] Jabari L, Gannoun H, Cayol J-L, Hedi A, Sakamoto M, et al (2012) *Macellibacteroides fermentans* gen. nov., sp. nov., a member of the family Porphyromonadaceae isolated from an upflow anaerobic filter treating abattoir wastewaters. International Journal of Systematic and Evolutionary Microbiology 62: 2522–2527. 22180609 10.1099/ijs.0.032508-0

[48] Dethlefsen L, Huse S, Sogin ML, Relman DA (2008) The Pervasive Effects of an Antibiotic on the Human Gut Microbiota, as Revealed by Deep 16S rRNA Sequencing. PLoS Biol 6: e280. 19018661 10.1371/journal.pbio.0060280 PMC2586385

[49] Ley RE, Turnbaugh PJ, Klein S, Gordon JI (2006) Microbial ecology: Human gut microbes associated with obesity. Nature 444: 1022–1023. 17183309 10.1038/4441022a

[50] Palmer C, Bik EM, DiGiulio DB, Relman DA, Brown PO (2007) Development of the Human Infant Intestinal Microbiota. PLoS Biol 5: e177. 17594176 10.1371/journal.pbio.0050177 PMC1896187

[51] Dethlefsen L, McFall-Ngai M, Relman DA (2007) An ecological and evolutionary perspective on human–microbe mutualism and disease. Nature 449: 811–818. 17943117 10.1038/nature06245 PMC9464033

