# Supplementary figures and images for: The Effect of Dietary Supplementation with Spent Cider Yeast on the Swine Distal Gut Microbiome
Source: PLoS One. 2013 Oct 9;8(10):e75714. doi: 10.1371/journal.pone.0075714 (PMC3794030; doi:10.1371/journal.pone.0075714)

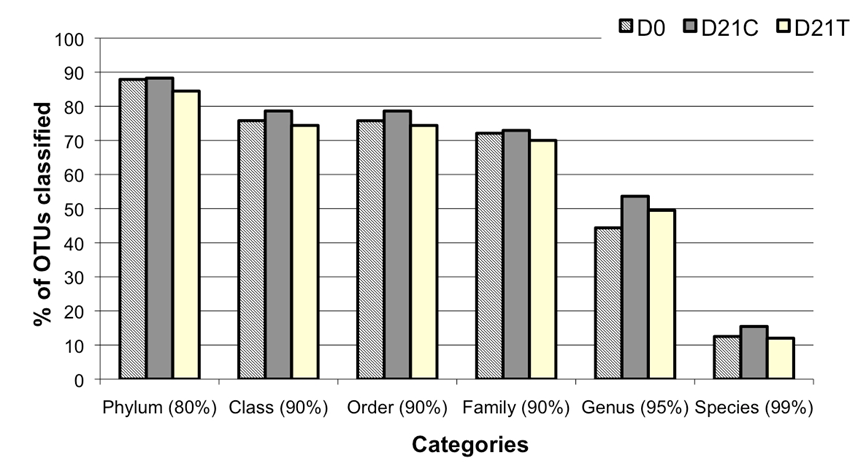

Supplement: Figure S1 — Percentages of sequences that are classified into OTU’s, using identity threshold of 80% for Phylum, 90% for Class, Order and Family, 95% for Genus and 99% for Species. (TIF) [file pone.0075714.s001.tif]

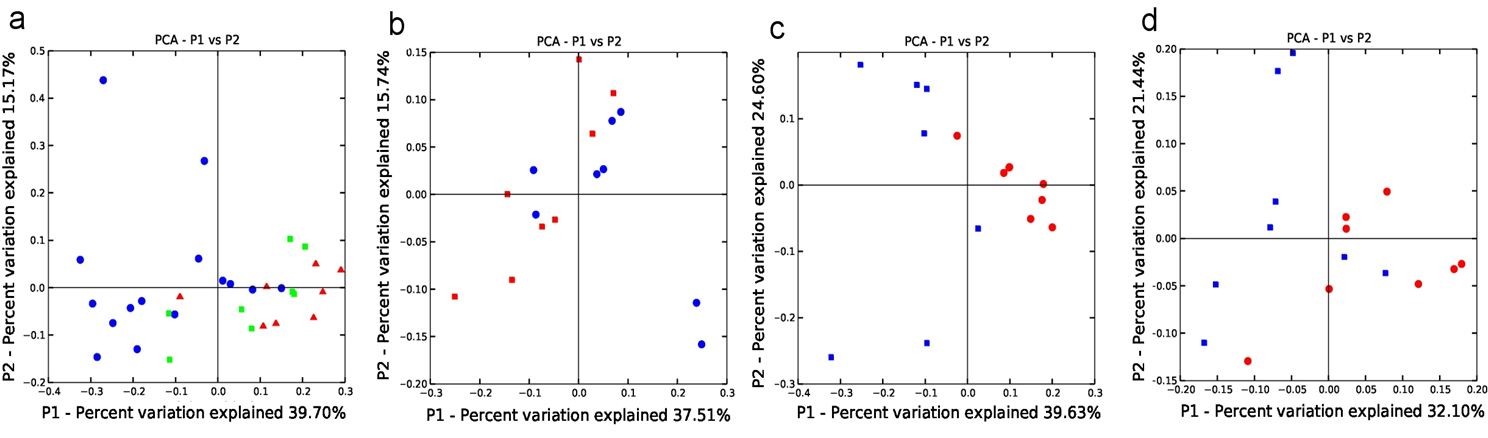

Supplement: Figure S2 — Weighted UniFrac principal component analysis. for a) control and treatment animals in day 0 (blue-circles), control animals in day 21 (green-squares) and treatment animals in day 21 (red-triangles) b) Control animals in day 0 (red-squares) and control animals in day 21 (blue-circle) c) Treatment animals in day 0 (blue-squares) and treatment animals in day 21 (red-circles) d) Control animals in day21 (blue-squares) and treatment animals in day 21 (red-circles). (TIF) [file pone.0075714.s002.tif]

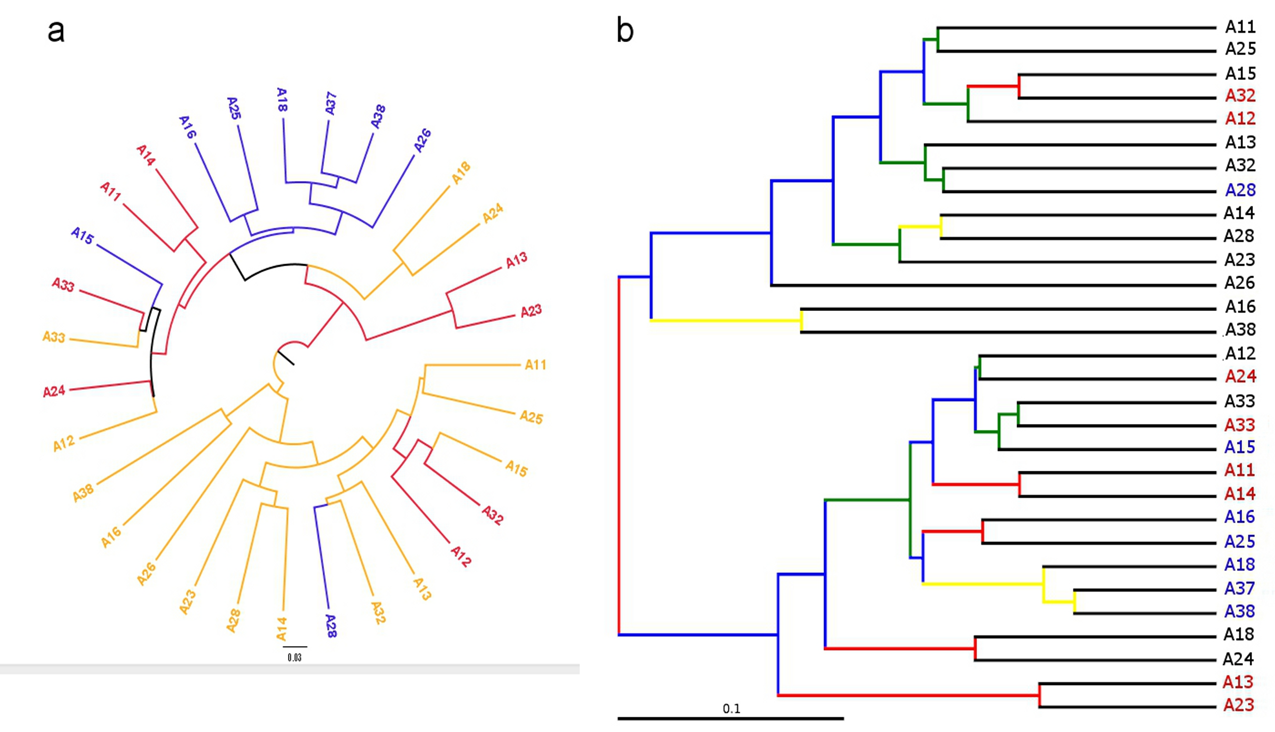

Supplement: Figure S3 — a) UPGMA clustering and Jackknifing for the weighted UniFrac data. For the UPGMA cladogram on the left: Orange colour represents animals at day 0; red for the control animals at day 21 and blue for the treatment animals at day 21. b) For the Jackknife supported tree layout the labels are coloured according to the group as: Black for animals in day 0; red for the control animals in day 21 and blue for treatment animals in day 21. The lines are coloured by the Jackknife supported percentages: Red for 75–100% support; Green for 50–75% support; Yellow for 25–50% support and Blue for <25% support. (TIF) [file pone.0075714.s003.tif]

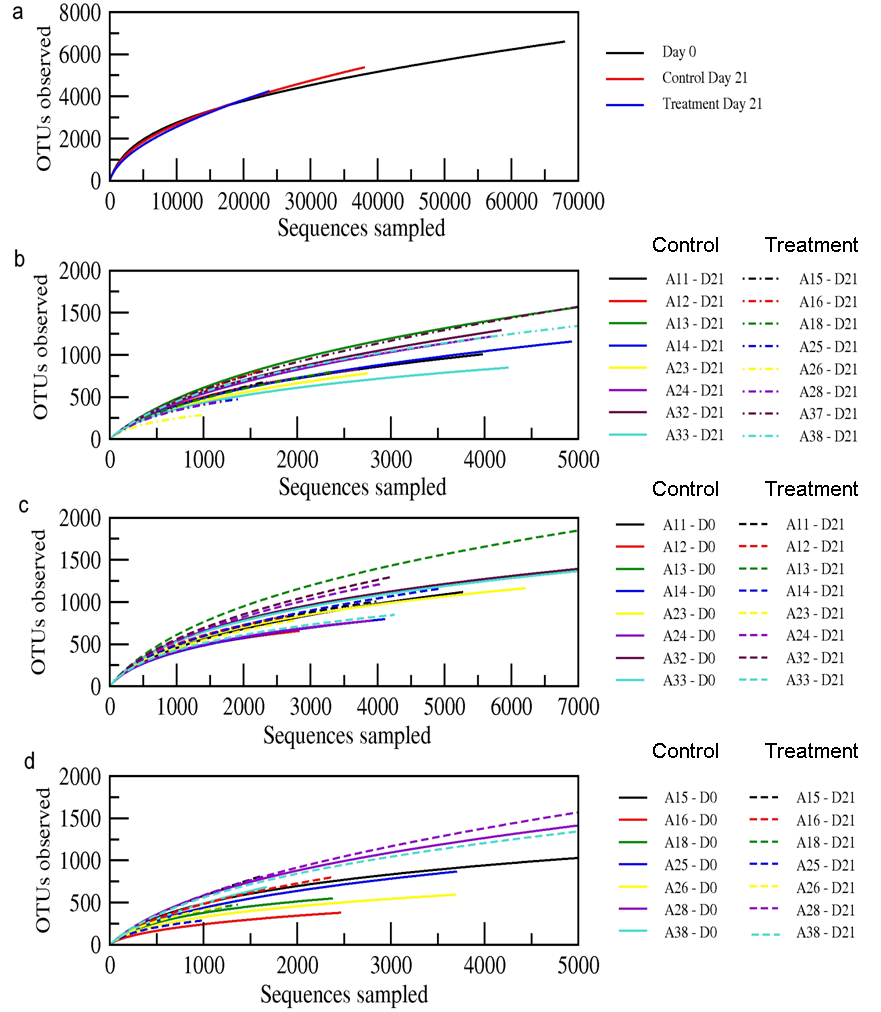

Supplement: Figure S4 — a) Rarefaction curves for the animals in day 0 (blue), control animals in day 21 (orange) and treatment animal in day 21 (green) for the 0.03 distance uniqueness values. b) For the control animals and treatment animals at day 21 (D21). Animal labels are consistent with the labels used in the supplementary table ST3. c) For the control animals at day 0 and day 21 (A-animal, C-control D-day 0 or 21) d) For the treatment animals at day 0 and day 21 (A-animal, T-treatment D-day 0 or 21). (TIF) [file pone.0075714.s004.tif]
