# Supplementary material for: The Effect of Dietary Supplementation with Spent Cider Yeast on the Swine Distal Gut Microbiome
Source: PLoS One. 2013 Oct 9;8(10):e75714. doi: 10.1371/journal.pone.0075714 (PMC3794030; doi:10.1371/journal.pone.0075714)
Supplement: Table S1 — Composition of diets fed to the pigs. (DOC) [file pone.0075714.s005.doc]

**Table S1**. Composition of diets fed to the pigs

|  | Control | | Treatment |
| --- | --- | --- | --- |
| **Ingredients** | Vigour | Startrite 88 | **Cider Yeast** |
| **Oil** | 8.0 | 9.5 | **4.8 %** |
| **Crude protein** | 22.5 | 24.0 | **21.5 %** |
| **Crude fibre** | 3.0 | 2.2 | **6.0 %** |
| **Crude ash** | 6.2 | 6.8 | **3.4 %** |
| **Lysine** | 1.5 | 1.65 | **-** |
| **Vitamin A** | 13000 | 13000 | **-** |
| **Vitamin D_3_** | 2000 | 2000 | **-** |
| **Vitamin E** | 200 | 250 | **-** |
| **Selenium** | 0.3 | 0.3 | **-** |
| **Dry matter** | - | - | **15.0 %** |
