# Supplementary material for: The Effect of Dietary Supplementation with Spent Cider Yeast on the Swine Distal Gut Microbiome
Source: PLoS One. 2013 Oct 9;8(10):e75714. doi: 10.1371/journal.pone.0075714 (PMC3794030; doi:10.1371/journal.pone.0075714)
Supplement: Table S2 — Effect of cider yeast on pig intake and growth performance. (DOC) [file pone.0075714.s006.doc]

**Table S2**. Effect of cider yeast on pig intake and growth performance

| Parameters | Control | Treatment | SEM | P-value |
| --- | --- | --- | --- | --- |
| Body weight at d 0 | 10.8 | 10.8 | 0.34 | 0.94 |
| **Stage 1, d 0 to 7** |  |  |  |  |
| DFI | 517 | 500 | 24.6 | 0.58 |
| ADG | 354 | 405 | 26.6 | 0.14 |
| FCE | 1.52 | 1.25 | 0.098 | 0.04 |
| Body weight at d 7 | 13.2 | 13.5 | 0.19 | 0.14 |
| **Stage 2, d 7 to 14** |  |  |  |  |
| DFI | 886 | 786 | 18.8 | 0.02 |
| ADG | 786 | 654 | 26.0 | 0.001 |
| FCE | 1.13 | 1.28 | 0.028 | 0.001 |
| Body weight at d 14 | 18.7 | 18.1 | 0.26 | 0.09 |
| **Stage 3, d 14 to 21** |  |  |  |  |
| DFI | 1054 | 1040 | 26.9 | 0.66 |
| ADG | 715 | 738 | 28.5 | 0.51 |
| FCE | 1.51 | 1.44 | 0.051 | 0.23 |
| **Stage 4, d 0 to 14** |  |  |  |  |
| DFI | 701 | 664 | 17.3 | 0.09 |
| ADG | 570 | 529 | 18.4 | 0.09 |
| FCE | 1.23 | 1.26 | 0.027 | 0.40 |
| Body weight at d 21 | 23.7 | 23.3 | 0.41 | 0.44 |
| **Stage 5, d 0 to 21** |  |  |  |  |
| DFI | 819 | 789 | 18.1 | 0.20 |
| ADG | 618 | 599 | 19.2 | 0.43 |
| FCE | 1.33 | 1.32 | 0.027 | 0.79 |
