# Supplementary material for: The Effect of Dietary Supplementation with Spent Cider Yeast on the Swine Distal Gut Microbiome
Source: PLoS One. 2013 Oct 9;8(10):e75714. doi: 10.1371/journal.pone.0075714 (PMC3794030; doi:10.1371/journal.pone.0075714)
Supplement: Table S3 — Amount of Cider yeast (ml) consumption by the animals during 21 days and its meal equivalent (g). (DOC) [file pone.0075714.s007.doc]

**Table** S**3** Amount of Cider yeast (ml) consumption by the animals during 21 days and its meal equivalent (g)

| Animal | 15 | 16 | 17 | 18 | 25 | 26 | 27 | 28 | 35 | 36 | 37 | 38 | Average | SD |
| --- | --- | --- | --- | --- | --- | --- | --- | --- | --- | --- | --- | --- | --- | --- |
| Day 0-7 |  |  |  |  |  |  |  |  |  |  |  |  |  |  |
| Total ml | 1781.67 | 2116 | 2317 | 2218 | 2191.7 | 2092.7 | 2227 | 2328 | 2035 | 2069.3 | 2337 | 2338 | 2171 | 164.2 |
| Meal equiv. (g) | 307.184 | 364.83 | 399.483 | 382.41 | 377.87 | 360.8 | 383.97 | 401.38 | 350.86 | 356.78 | 402.93 | 403.1 | 374 | 28.3 |
| Day 7-14 |  |  |  |  |  |  |  |  |  |  |  |  |  |  |
| Total ml | 2266.67 | 2600 | 2900 | 2800 | 2866.7 | 2766.7 | 2900 | 2900 | 2600 | 2733.3 | 2900 | 2900 | 2761 | 192.2 |
| Meal equiv. (g) | 390.805 | 448.28 | 500 | 482.76 | 494.25 | 477.01 | 500 | 500 | 448.28 | 471.26 | 500 | 500 | 476 | 33.1 |
| Day 14-21 |  |  |  |  |  |  |  |  |  |  |  |  |  |  |
| Total ml | 2566.67 | 3100 | 3000 | 3400 | 3366.7 | 3166.7 | 3500 | 3000 | 2100 | 2633.3 | 3500 | 3500 | 3069 | 442.8 |
| Meal equiv. (g) | 442.529 | 534.48 | 517.241 | 586.21 | 580.46 | 545.98 | 603.45 | 517.24 | 362.07 | 454.02 | 603.45 | 603.45 | 529 | 76.3 |
| Day 0-21 |  |  |  |  |  |  |  |  |  |  |  |  |  |  |
| Total cider consumption over 21 days | 6615 | 14129 | 14716.5 | 14983 | 14930 | 14597 | 15111 | 14729 | 13234 | 14031 | 15240 | 15241 | 13963 | 2386.9 |
| Meal equiv. (g) over 21 days | 1140.52 | 1347.6 | 1416.72 | 1451.4 | 1452.6 | 1383.8 | 1487.4 | 1418.6 | 1161.2 | 1282.1 | 1506.4 | 1506.6 | 1380 | 125.3 |
