# Supplementary material for: The Effect of Dietary Supplementation with Spent Cider Yeast on the Swine Distal Gut Microbiome
Source: PLoS One. 2013 Oct 9;8(10):e75714. doi: 10.1371/journal.pone.0075714 (PMC3794030; doi:10.1371/journal.pone.0075714)
Supplement: Table S4 — Sample richness estimators, Shannon diversity indices, Chao1 richness, and Good’s coverage for the sequences classified at 95% level of similarity. Shannon indices for the samples estimated from normalized and unnormalized sequences from PANGEA. (DOC) [file pone.0075714.s008.doc]

**Table S4**: Sample richness estimators, Shannon diversity indices, Chao1 richness, and Good’s coverage for the sequences classified at 95% level of similarity. Shannon indices for the samples estimated from normalized and unnormalized sequences from PANGEA.

| Sample identity | Description | Total number of sequences | Number of seqs classified at 95% (genus level) | % of seqs classified at 95% | Good’s coverage | Simpson | ACE | Chao1 | Shannon  original | Shannon  normalised |
| --- | --- | --- | --- | --- | --- | --- | --- | --- | --- | --- |
| Day 0 group | D0 C & T | 73359 | 37335 | 50.90 |  |  |  |  |  |  |
| A11 | D0 C | 5276 | 3803 | 72.08 | 94.0 | 0.011 | 1527.1 | 1184.2 | 4.8 | 4.22 |
| A12 | D0 C | 2833 | 1613 | 56.94 | 95.8 | 0.008 | 506.1 | 534.3 | 4.77 | 4.32 |
| A13 | D0 C | 6936 | 3930 | 56.66 | 94.1 | 0.003 | 1567.3 | 1728.8 | 4.85 | 4.37 |
| A14 | D0 C | 4084 | 1608 | 39.37 | 95.2 | 0.008 | 949.1 | 812.5 | 4.57 | 4.21 |
| A15 | D0 T | 5438 | 2097 | 38.56 | 96.1 | 0.004 | 884.4 | 924.8 | 4.79 | 4.34 |
| A16 | D0 T | 2456 | 755 | 30.74 | 94.8 | 0.034 | 649.7 | 451.6 | 2.70 | 2.48 |
| A18 | D0 T | 2380 | 796 | 33.44 | 95.2 | 0.009 | 463.0 | 493.8 | 4.18 | 3.84 |
| A23 | D0 C | 6210 | 3521 | 56.69 | 96.2 | 0.005 | 1123.3 | 1006.3 | 4.90 | 4.50 |
| A24 | D0 C | 3911 | 1465 | 37.46 | 96.3 | 0.007 | 623.7 | 667.5 | 4.18 | 3.81 |
| A25 | D0 T | 3672 | 2698 | 73.47 | 92.9 | 0.007 | 1264.8 | 1029.7 | 5.10 | 4.64 |
| A26 | D0 T | 3689 | 3141 | 85.15 | 96.5 | 0.011 | 617.1 | 538.0 | 4.51 | 4.03 |
| A28 | D0 T | 7037 | 4071 | 57.85 | 93.0 | 0.003 | 2500.9 | 2007.7 | 5.39 | 4.72 |
| A32 | D0 C | 7501 | 4346 | 57.94 | 96.7 | 0.004 | 1071.7 | 1103.7 | 5.10 | 4.51 |
| A33 | D0 C | 7773 | 2713 | 34.90 | 96.6 | 0.004 | 1282.6 | 1183.8 | 4.43 | 4.09 |
| A37 | D0 T | 0 | 0 | 0 |  |  |  |  | 0.0 | 0.0 |
| A38 | D0 T | 4163 | 778 | 18.69 | 94.4 | 0.006 | 1115.8 | 976.6 | 4.22 | 4.00 |
| Control group | D21 C | 40934 | 16823 | 41.09 |  |  |  |  |  |  |
| A11 | D21 C | 3980 | 1104 | 27.74 | 93.0 | 0.004 | 1370.7 | 1200.2 | 4.22 | 3.97 |
| A12 | D21 C | 2627 | 1103 | 41.99 | 90.4 | 0.005 | 1094.8 | 1008.4 | 4.61 | 4.30 |
| A13 | D21 C | 14146 | 6389 | 45.16 | 41.3 | 0.0003 | 3976.1 | 4106.1 | 4.69 | 4.17 |
| A14 | D21 C | 4927 | 1605 | 32.58 | 93.3 | 0.005 | 1561.9 | 1450.5 | 4.19 | 3.95 |
| A23 | D21 C | 2752 | 1255 | 45.60 | 91.1 | 0.007 | 1173.7 | 1012.1 | 4.42 | 4.14 |
| A24 | D21 C | 4070 | 2069 | 50.84 | 91.0 | 0.004 | 1176.4 | 1464.7 | 5.00 | 4.51 |
| A32 | D21 C | 4179 | 2350 | 56.23 | 89.2 | 0.004 | 2195.3 | 1859.4 | 5.05 | 4.57 |
| A33 | D21 C | 4253 | 948 | 22.29 | 95.9 | 0.006 | 703.3 | 754.9 | 3.92 | 3.70 |
| Treatment group (CY) | D21 T | 25409 | 11307 | 44.50 |  |  |  |  |  |  |
| A15 | D21 T | 1674 | 742 | 44.32 | 81.9 | 0.007 | 1778.4 | 1115.1 | 4.65 | 4.37 |
| A16 | D21 T | 1601 | 846 | 52.84 | 74.1 | 0.004 | 2737.8 | 1540.9 | 4.95 | 4.59 |
| A18 | D21 T | 2404 | 1180 | 49.08 | 86.4 | 0.006 | 1890.1 | 1276.5 | 4.61 | 4.32 |
| A25 | D21 T | 1365 | 900 | 65.93 | 90.7 | 0.006 | 467.0 | 468.6 | 4.86 | 4.67 |
| A26 | D21 T | 1014 | 275 | 27.12 | 92.6 | 0.015 | 278.1 | 331.7 | 3.57 | 3.37 |
| A28 | D21 T | 1189 | 666 | 56.01 | 89.8 | 0.006 | 442.7 | 431.1 | 4.92 | 4.66 |
| A37 | D21 T | 6762 | 2674 | 39.54 | 92.8 | 0.004 | 2423.6 | 1848.0 | 4.53 | 4.12 |
| A38 | D21 T | 9400 | 4024 | 42.81 | 95.3 | 0.003 | 2106.6 | 1799.0 | 4.58 | 4.25 |
