# Supplementary material for: The Effect of Dietary Supplementation with Spent Cider Yeast on the Swine Distal Gut Microbiome
Source: PLoS One. 2013 Oct 9;8(10):e75714. doi: 10.1371/journal.pone.0075714 (PMC3794030; doi:10.1371/journal.pone.0075714)
Supplement: Table S5 — Comparison of taxonomic groups between the treatment (CY) and control groups at day 21. Using modified Chi-square test with false discovery rate (FDR). (DOC) [file pone.0075714.s009.doc]

**Table S5:** Comparison of taxonomic groups between the treatment (CY) and control groups at day 21. Using modified Chi-square test with false discovery rate (FDR).

| O.T.U. | p-value | FDR |
| --- | --- | --- |
| **PHYLUM** |  |  |
| Bacteroidetes | 0 | 0.003 |
| Chlamydiae | 0 | 0.007 |
| Fibrobacteres | 0 | 0.01 |
| Firmicutes | 0 | 0.013 |
| Proteobacteria | 0 | 0.017 |
| Spirochaetes | 0 | 0.02 |
| Tenericutes | 0.018 | 0.023 |
| Elusimicrobia | 0.165 | 0.027 |
| Synergistetes | 0.296 | 0.03 |
| Actinobacteria | 0.507 | 0.033 |
| Lentisphaerae | 0.534 | 0.037 |
| Acidobacteria | 1 | 0.04 |
| Deferribacteres | 1 | 0.043 |
| Fusobacteria | 1 | 0.047 |
| Streptophyta | 1 | 0.05 |
| **CLASS** |  |  |
| Bacilli | 0 | 0.002 |
| Bacteroidia | 0 | 0.005 |
| Betaproteobacteria | 0 | 0.007 |
| Chlamydiae | 0 | 0.01 |
| Clostridia | 0 | 0.012 |
| Fibrobacteres | 0 | 0.014 |
| Gammaproteobacteria | 0 | 0.017 |
| Spirochaetes | 0 | 0.019 |
| Elusimicrobia | 0.166 | 0.021 |
| Deltaproteobacteria | 0.276 | 0.024 |
| Synergistia | 0.295 | 0.026 |
| Erysipelotrichi | 0.296 | 0.029 |
| Mollicutes | 0.302 | 0.031 |
| Actinobacteria | 0.506 | 0.04 |
| Alphaproteobacteria | 0.656 | 0.043 |
| Deferribacteres | 1 | 0.045 |
| Epsilonproteobacteria | 1 | 0.048 |
| Fusobacteria | 1 | 0.05 |
| **ORDER** |  |  |
| Aeromonadales | 0 | 0.002 |
| Bacteroidales | 0 | 0.003 |
| Burkholderiales | 0 | 0.005 |
| Chlamydiales | 0 | 0.007 |
| Clostridiales | 0 | 0.009 |
| Enterobacteriales | 0 | 0.01 |
| Fibrobacterales | 0 | 0.012 |
| Lactobacillales | 0 | 0.014 |
| Pasteurellales | 0 | 0.016 |
| Spirochaetales | 0 | 0.017 |
| Syntrophobacterales | 0.052 | 0.019 |
| Anaeroplasmatales | 0.055 | 0.021 |
| Desulfovibrionales | 0.123 | 0.022 |
| Xanthomonadales | 0.139 | 0.024 |
| Desulfuromonadales | 0.141 | 0.026 |
| Bacillales | 0.162 | 0.028 |
| Elusimicrobiales | 0.164 | 0.029 |
| Pseudomonadales | 0.292 | 0.031 |
| Erysipelotrichales | 0.293 | 0.033 |
| Synergistales | 0.295 | 0.034 |
| Coriobacteriales | 0.505 | 0.041 |
| Rhodobacterales | 0.661 | 0.043 |
| Acholeplasmatales | 1 | 0.045 |
| Campylobacterales | 1 | 0.047 |
| Deferribacterales | 1 | 0.048 |
| Fusobacteriales | 1 | 0.05 |
| **FAMILY** |  |  |
| Alcaligenaceae | 0 | 0.001 |
| Bacteroidaceae | 0 | 0.002 |
| Chlamydiaceae | 0 | 0.003 |
| Clostridiaceae | 0 | 0.004 |
| Enterobacteriaceae | 0 | 0.005 |
| Fibrobacteraceae | 0 | 0.006 |
| Lactobacillaceae | 0 | 0.007 |
| Oscillospiraceae | 0 | 0.008 |
| Pasteurellaceae | 0 | 0.009 |
| Porphyromonadaceae | 0 | 0.01 |
| Prevotellaceae | 0 | 0.011 |
| Ruminococcaceae | 0 | 0.012 |
| Spirochaetaceae | 0 | 0.013 |
| Succinivibrionaceae | 0 | 0.014 |
| Veillonellaceae | 0 | 0.015 |
| Peptococcaceae | 0.001 | 0.016 |
| Syntrophaceae | 0.053 | 0.017 |
| Anaeroplasmataceae | 0.055 | 0.018 |
| Lachnospiraceae | 0.063 | 0.019 |
| Desulfovibrionaceae | 0.092 | 0.02 |
| Geobacteraceae | 0.144 | 0.021 |
| Xanthomonadaceae | 0.145 | 0.022 |
| Elusimicrobiaceae | 0.164 | 0.023 |
| Rikenellaceae | 0.166 | 0.024 |
| Leuconostocaceae | 0.189 | 0.026 |
| Eubacteriaceae | 0.228 | 0.027 |
| Streptococcaceae | 0.294 | 0.028 |
| Synergistaceae | 0.294 | 0.029 |
| Moraxellaceae | 0.296 | 0.03 |
| Erysipelotrichaceae | 0.356 | 0.031 |
| Clostridiales Family XVI. Incertae Sedis | 0.378 | 0.032 |
| Alcaligenaceae | 0.42 | 0.035 |
| Bacteroidaceae | 0.506 | 0.036 |
| Chlamydiaceae | 0.526 | 0.037 |
| Clostridiaceae | 0.532 | 0.038 |
| Enterobacteriaceae | 0.532 | 0.039 |
| Fibrobacteraceae | 0.56 | 0.04 |
| Lactobacillaceae | 0.649 | 0.041 |
| Oscillospiraceae | 0.656 | 0.042 |
| Pasteurellaceae | 0.664 | 0.043 |
| Porphyromonadaceae | 0.706 | 0.044 |
| Prevotellaceae | 0.88 | 0.045 |
| Ruminococcaceae | 1 | 0.046 |
| Spirochaetaceae | 1 | 0.047 |
| Succinivibrionaceae | 1 | 0.048 |
| Veillonellaceae | 1 | 0.049 |
| Peptococcaceae | 1 | 0.05 |
| **GENUS** |  |  |
| Acidaminococcus | 0 | 0 |
| Actinobacillus | 0 | 0.001 |
| Alkaliphilus | 0 | 0.001 |
| Anaerovibrio | 0 | 0.002 |
| Bacteroides | 0 | 0.002 |
| Butyricicoccus | 0 | 0.003 |
| Catenibacterium | 0 | 0.003 |
| Chlamydia | 0 | 0.003 |
| Clostridium | 0 | 0.004 |
| Escherichia | 0 | 0.004 |
| Haemophilus | 0 | 0.005 |
| Lachnospira | 0 | 0.005 |
| Lactobacillus | 0 | 0.005 |
| Megasphaera | 0 | 0.006 |
| Mitsuokella | 0 | 0.006 |
| Porphyromonadaceae bacterium DJF B175 | 0 | 0.007 |
| Prevotella | 0 | 0.007 |
| Ruminococcus | 0 | 0.008 |
| Salmonella | 0 | 0.008 |
| Selenomonas | 0 | 0.008 |
| Subdoligranulum | 0 | 0.009 |
| Succinivibrio | 0 | 0.009 |
| Sutterella | 0 | 0.01 |
| Veillonellaceae bacterium WK011 | 0 | 0.01 |
| Anaerobiospirillum | 0.001 | 0.01 |
| Clostridiaceae bacterium DJF VP56 | 0.001 | 0.011 |
| Eubacterium biforme | 0.001 | 0.011 |
| Prevotellaceae bacterium DJF VR15 | 0.001 | 0.012 |
| Lachnobacterium | 0.002 | 0.012 |
| Prevotellaceae bacterium DJF RP84 | 0.005 | 0.012 |
| Clostridiaceae bacterium DJF B063 | 0.007 | 0.013 |
| Coprococcus | 0.011 | 0.013 |
| Clostridiaceae bacterium SN021 | 0.015 | 0.014 |
| Desulfovibrio | 0.016 | 0.014 |
| Leuconostoc | 0.016 | 0.015 |
| Actinobacillus rossii | 0.018 | 0.015 |
| Clostridiaceae bacterium 37-7-2Cl | 0.018 | 0.015 |
| Lachnospiraceae bacterium DJF VP52 | 0.018 | 0.016 |
| Clostridiaceae bacterium SH021 | 0.024 | 0.016 |
| Eubacterium | 0.024 | 0.017 |
| Lachnospiraceae bacterium DJF CR52 | 0.04 | 0.017 |
| Treponema | 0.098 | 0.018 |
| Lachnospiraceae bacterium DJF VP30 | 0.101 | 0.018 |
| Phascolarctobacterium | 0.106 | 0.018 |
| Lachnospiraceae bacterium DJF VP18k1 | 0.107 | 0.019 |
| Prevotellaceae bacterium P4P 62 P1 | 0.109 | 0.019 |
| Roseburia | 0.131 | 0.02 |
| Stenotrophomonas | 0.135 | 0.02 |
| Blautia | 0.138 | 0.02 |
| Turicibacter | 0.139 | 0.021 |
| Acetitomaculum | 0.156 | 0.021 |
| Anaerostipes | 0.166 | 0.022 |
| Clostridiaceae bacterium WN011 | 0.168 | 0.022 |
| Prevotellaceae bacterium DJF RP17 | 0.188 | 0.023 |
| Clostridiaceae bacterium DJF VR76 | 0.265 | 0.023 |
| Oscillospiraceae bacterium NML 061048 | 0.276 | 0.023 |
| Eubacteriaceae bacterium DJF VR85 | 0.292 | 0.024 |
| Lachnospiraceae bacterium DJF VR44 | 0.298 | 0.024 |
| Pyramidobacter | 0.301 | 0.025 |
| Acinetobacter | 0.301 | 0.025 |
| Lactococcus | 0.302 | 0.025 |
| Clostridiaceae bacterium FH052 | 0.302 | 0.026 |
| Elusimicrobium | 0.303 | 0.026 |
| Citrobacter | 0.303 | 0.027 |
| Clostridiaceae bacterium NML 061030 | 0.304 | 0.027 |
| Bisgaard Taxon 10 | 0.304 | 0.028 |
| Shigella | 0.305 | 0.028 |
| Clostridiaceae bacterium K10 | 0.341 | 0.028 |
| Clostridiaceae bacterium DJF LS13 | 0.35 | 0.029 |
| Clostridiaceae bacterium LEMV61 | 0.361 | 0.029 |
| Coriobacteriaceae bacterium WAL 18889 | 0.368 | 0.03 |
| Eubacteriaceae bacterium PEH A | 0.368 | 0.03 |
| Lactobacillus vitulinus | 0.37 | 0.03 |
| Oribacterium | 0.37 | 0.031 |
| Sorghum | 0.37 | 0.031 |
| Herbaspirillum | 0.37 | 0.032 |
| Oenococcus | 0.371 | 0.032 |
| Anaeroglobus | 0.371 | 0.032 |
| Turicibacter | 0.372 | 0.033 |
| Acetitomaculum | 0.373 | 0.034 |
| Anaerostipes | 0.373 | 0.034 |
| Clostridiaceae bacterium WN011 | 0.42 | 0.035 |
| Prevotellaceae bacterium DJF RP17 | 0.423 | 0.035 |
| Clostridiaceae bacterium DJF VR76 | 0.48 | 0.035 |
| Oscillospiraceae bacterium NML 061048 | 0.481 | 0.036 |
| Eubacteriaceae bacterium DJF VR85 | 0.529 | 0.036 |
| Lachnospiraceae bacterium DJF VR44 | 0.533 | 0.037 |
| Pyramidobacter | 0.534 | 0.037 |
| Acinetobacter | 0.536 | 0.038 |
| Lactococcus | 0.537 | 0.038 |
| Clostridiaceae bacterium FH052 | 0.609 | 0.038 |
| Elusimicrobium | 0.611 | 0.039 |
| Citrobacter | 0.628 | 0.039 |
| Clostridiaceae bacterium NML 061030 | 0.632 | 0.04 |
| Bisgaard Taxon 10 | 0.777 | 0.04 |
| Shigella | 0.812 | 0.04 |
| Clostridiaceae bacterium K10 | 0.841 | 0.041 |
| Clostridiaceae bacterium DJF LS13 | 0.843 | 0.041 |
| Clostridiaceae bacterium LEMV61 | 0.88 | 0.042 |
| Coriobacteriaceae bacterium WAL 18889 | 0.893 | 0.042 |
| Eubacteriaceae bacterium PEH A | 1 | 0.042 |
| Lactobacillus vitulinus | 1 | 0.043 |
| Oribacterium | 1 | 0.043 |
| Sorghum | 1 | 0.044 |
| Herbaspirillum | 1 | 0.044 |
| Oenococcus | 1 | 0.045 |
| Anaeroglobus | 1 | 0.045 |
| Anaerosporobacter | 1 | 0.046 |
| Pelosinus | 1 | 0.046 |
| Parabacteroides | 1 | 0.047 |
| Sporobacter | 1 | 0.048 |
| Pseudobutyrivibrio | 1 | 0.048 |
| Schwartzia | 1 | 0.048 |
| Guggenheimella | 1 | 0.049 |
| Dorea | 1 | 0.049 |
| Victivallis | 1 | 0.05 |
| **SPECIES** |  |  |
| Acidaminococcus fermentans | 0 | 0 |
| anaerobic bacterium A107 | 0 | 0.001 |
| bacterium mpn-isolate group 3 | 0 | 0.001 |
| Catenibacterium mitsuokai | 0 | 0.001 |
| Haemophilus parahaemolyticus | 0 | 0.002 |
| Lactobacillus amylovorus | 0 | 0.002 |
| Lactobacillus johnsonii | 0 | 0.002 |
| Lactobacillus reuteri | 0 | 0.003 |
| Megasphaera elsdenii | 0 | 0.003 |
| Mitsuokella multacida | 0 | 0.003 |
| Porphyromonadaceae bacterium DJF B175 | 0 | 0.004 |
| Prevotella sp. DJFRP53 | 0 | 0.004 |
| Roseburia hominis | 0 | 0.004 |
| Ruminococcus bromii | 0 | 0.005 |
| swine fecal bacterium SD-Pec10 | 0 | 0.005 |
| Prevotella sp. RS2 | 0.001 | 0.005 |
| bacterium mpn-isolate group 17 | 0.002 | 0.006 |
| Megasphaera sp. TrE9262 | 0.006 | 0.006 |
| Coprococcus catus | 0.012 | 0.007 |
| Phascolarctobacterium sp. YIT 12068 | 0.012 | 0.007 |
| Succinivibrio dextrinosolvens | 0.013 | 0.007 |
| Selenomonas ruminantium | 0.019 | 0.008 |
| Roseburia sp. 11SE39 | 0.02 | 0.008 |
| bacterium 8-gw1-8 | 0.021 | 0.008 |
| bacterium mpn-isolate group 2 | 0.023 | 0.009 |
| Lachnospiraceae bacterium DJF CR52 | 0.026 | 0.009 |
| Roseburia inulinivorans | 0.03 | 0.009 |
| Escherichia coli | 0.037 | 0.01 |
| Prevotella sp. DJF LS16 | 0.038 | 0.01 |
| Selenomonas sp. Ycb08 | 0.038 | 0.01 |
| Prevotellaceae bacterium DJF RP84 | 0.039 | 0.011 |
| Prevotellaceae bacterium DJF VR15 | 0.045 | 0.011 |
| Clostridiaceae bacterium DJF VP56 | 0.05 | 0.011 |
| Salmonella enterica | 0.051 | 0.012 |
| Lachnospiraceae bacterium DJF VP18k1 | 0.052 | 0.012 |
| Pseudobutyrivibrio xylanivorans | 0.059 | 0.012 |
| Chlamydia suis | 0.09 | 0.013 |
| Oxalobacter formigenes | 0.104 | 0.013 |
| Sutterella sp. YIT 12072 | 0.111 | 0.013 |
| Stenotrophomonas rhizophila | 0.113 | 0.014 |
| Actinobacillus porcinus | 0.114 | 0.014 |
| swine fecal bacterium RF3F-Cel1 | 0.115 | 0.014 |
| Faecalibacterium sp. DJF VR20 | 0.13 | 0.015 |
| Eubacterium rectale | 0.141 | 0.015 |
| Lactobacillus crispatus | 0.176 | 0.015 |
| Eubacterium eligens | 0.178 | 0.016 |
| Bacteroides galacturonicus | 0.184 | 0.016 |
| Mitsuokella sp. TM-10 | 0.204 | 0.016 |
| Anaerobiospirillum succiniciproducens | 0.211 | 0.017 |
| Actinobacillus minor | 0.236 | 0.017 |
| Clostridiaceae bacterium DJF LS40 | 0.243 | 0.017 |
| Ruminococcus obeum | 0.306 | 0.018 |
| unidentified eubacterium clone BSV07 | 0.309 | 0.018 |
| Desulfovibrio piger | 0.31 | 0.018 |
| Prevotella sp. DJF B116 | 0.318 | 0.019 |
| Citrobacter werkmanii | 0.328 | 0.019 |
| Clostridiales bacterium A2-162 | 0.329 | 0.02 |
| Acidaminococcus intestini | 0.33 | 0.02 |
| Treponema berlinense | 0.332 | 0.02 |
| butyrate-producing bacterium Ph07AY04 | 0.333 | 0.021 |
| Campylobacter coli | 0.333 | 0.021 |
| Coriobacteriaceae bacterium WAL 18889 | 0.333 | 0.021 |
| Eubacterium desmolans | 0.333 | 0.022 |
| Herbaspirillum seropedicae | 0.335 | 0.022 |
| Lactobacillus sp. HJ2 | 0.336 | 0.022 |
| Eubacterium siraeum | 0.337 | 0.023 |
| swine manure bacterium 37-2 | 0.337 | 0.023 |
| Vitis vinifera | 0.337 | 0.023 |
| Sorghum bicolor | 0.338 | 0.024 |
| Actinobacillus rossii | 0.339 | 0.024 |
| ubacterium sp. C124b | 0.339 | 0.024 |
| Lactobacillus vitulinus | 0.339 | 0.025 |
| Pediococcus parvulus | 0.339 | 0.025 |
| methanogenic archaeon LGM-AFM01 | 0.341 | 0.025 |
| Bacteroides pectinophilus | 0.428 | 0.026 |
| Helicobacter equorum | 0.447 | 0.026 |
| Clostridiaceae bacterium DJF VR76 | 0.448 | 0.026 |
| Parasutterella sp. YIT 12071 | 0.454 | 0.027 |
| butyrate-producing bacterium T1-815 | 0.552 | 0.027 |
| Bisgaard Taxon 10 | 0.553 | 0.027 |
| Lactobacillus sp. KC36a | 0.553 | 0.028 |
| Bacteroides thetaiotaomicron | 0.554 | 0.028 |
| Lachnospiraceae bacterium DJF VR44 | 0.555 | 0.028 |
| bacterium RA2074 | 0.608 | 0.029 |
| Prevotella sp. BI-42 | 0.609 | 0.029 |
| Ruminococcus callidus | 0.667 | 0.029 |
| butyrate-producing bacterium PH07AY3 | 0.67 | 0.03 |
| Lachnospiraceae bacterium DJF VP52 | 0.67 | 0.03 |
| Allisonella histaminiformans | 0.672 | 0.03 |
| Coprococcus comes | 0.695 | 0.031 |
| Faecalibacterium prausnitzii | 0.711 | 0.031 |
| human intestinal bacterium PUE | 0.725 | 0.032 |
| Prevotellaceae bacterium DJF RP17 | 0.736 | 0.032 |
| butyrate-producing bacterium A2-166 | 0.773 | 0.032 |
| Eubacterium sp. cL-10-1-3 | 0.794 | 0.033 |
| bacterium ASF500 | 0.817 | 0.033 |
| Prevotellaceae bacterium DJF LS10 | 0.844 | 0.033 |
| Acidaminococcus sp. DJF RP55 | 1 | 0.034 |
| Acinetobacter baumannii | 1 | 0.034 |
| Acinetobacter brisoui | 1 | 0.034 |
| Alistipes indistinctus | 1 | 0.035 |
| Anaerovibrio lipolyticus | 1 | 0.035 |
| bacterium 'New Zealand D' | 1 | 0.035 |
| bacterium GIST-YKR71 | 1 | 0.036 |
| bacterium ii1269 | 1 | 0.036 |
| bacterium RA2108 | 1 | 0.036 |
| Bacteroides plebeius | 1 | 0.037 |
| Bacteroides sp. DSM 12148 | 1 | 0.037 |
| butyrate-producing bacterium PH05YB02 | 1 | 0.037 |
| Campylobacter jejuni | 1 | 0.038 |
| Clostridiaceae bacterium DJF LS13 | 1 | 0.038 |
| Clostridiales bacterium W060 | 1 | 0.038 |
| Clostridium butyricum | 1 | 0.039 |
| Clostridium sp. MLG661 | 1 | 0.039 |
| Clostridium sporosphaeroides | 1 | 0.039 |
| Desulfovibrio sp. LNB1 | 1 | 0.04 |
| Escherichia fergusonii | 1 | 0.04 |
| Escherichia sp. 21CR | 1 | 0.04 |
| Fibrobacter intestinalis | 1 | 0.041 |
| Firmicutes bacterium DJF VR50 | 1 | 0.041 |
| Firmicutes bacterium VNs39 | 1 | 0.041 |
| Haemophilus parasuis | 1 | 0.042 |
| Lachnospiraceae bacterium 28-4 | 1 | 0.042 |
| Lachnospiraceae bacterium DJF RP14 | 1 | 0.042 |
| Lachnospiraceae bacterium DJF VP30 | 1 | 0.043 |
| Lactobacillus delbrueckii | 1 | 0.043 |
| Lactobacillus sp. ID9203 | 1 | 0.043 |
| Lactobacillus vaginalis | 1 | 0.044 |
| Leuconostoc citreum | 1 | 0.044 |
| Leuconostoc garlicum | 1 | 0.045 |
| Leuconostoc mesenteroides | 1 | 0.045 |
| Mitsuokella sp. DJF VR02k1 | 1 | 0.045 |
| Parabacteroides merdae | 1 | 0.046 |
| Pediococcus ethanolidurans | 1 | 0.046 |
| Phaseolus vulgaris | 1 | 0.046 |
| rape rhizosphere bacterium F25 | 1 | 0.047 |
| Ruminococcus sp. DJF VR67 | 1 | 0.047 |
| Ruminococcus sp. Eg2 | 1 | 0.047 |
| Shigella boydii | 1 | 0.048 |
| Subdoligranulum sp. DJF VR33k2 | 1 | 0.048 |
| Sutterella stercoricanis | 1 | 0.048 |
| Swine fecal bacterium RF3G-Cel1 | 1 | 0.049 |
| Treponema porcinum | 1 | 0.049 |
| Turicibacter sanguinis | 1 | 0.049 |
| unidentified bacterium ZF5 | 1 | 0.05 |
| Victivallis vadensis | 1 | 0.05 |
